# Multi-omic signatures of sarcoidosis and progression in bronchoalveolar lavage cells

**DOI:** 10.1186/s12931-024-02919-7

**Published:** 2024-07-30

**Authors:** Iain R. Konigsberg, Nancy W. Lin, Shu-Yi Liao, Cuining Liu, Kristyn MacPhail, Margaret M. Mroz, Elizabeth Davidson, Clara I. Restrepo, Sunita Sharma, Li Li, Lisa A. Maier, Ivana V. Yang

**Affiliations:** 1 Department of Biomedical Informatics, School of Medicine, University of Colorado - Anschutz Medical Campus, Aurora, CO USA; 2 Division of Environmental and Occupational Health Sciences, Department of Medicine, National Jewish Health, Denver, CO USA; 3 Division of Pulmonary and Critical Care Sciences, Department of Medicine, School of Medicine, University of Colorado - Anschutz Medical Campus, Aurora, CO USA; 4 Division of Pulmonary and Critical Care Medicine, Department of Medicine, Johns Hopkins University, Baltimore, MD USA; 5 Department of Environmental and Occupational Health, Colorado School of Public Health, Aurora, CO USA; 6 Department of Biostatistics and Informatics, Colorado School of Public Health, Aurora, CO USA

**Keywords:** Sarcoidosis, Epigenetics, Multi-omics, DNA methylation, Gene expression, microRNA

## Abstract

**Background:**

Sarcoidosis is a heterogeneous granulomatous disease with no accurate biomarkers of disease progression. Therefore, we profiled and integrated the DNA methylome, mRNAs, and microRNAs to identify molecular changes associated with sarcoidosis and disease progression that might illuminate underlying mechanisms of disease and potential biomarkers.

**Methods:**

Bronchoalveolar lavage cells from 64 sarcoidosis subjects and 16 healthy controls were used. DNA methylation was profiled on Illumina HumanMethylationEPIC arrays, mRNA by RNA-sequencing, and miRNAs by small RNA-sequencing. Linear models were fit to test for effect of sarcoidosis diagnosis and progression phenotype, adjusting for age, sex, smoking, and principal components of the data. We built a supervised multi-omics model using a subset of features from each dataset.

**Results:**

We identified 1,459 CpGs, 64 mRNAs, and five miRNAs associated with sarcoidosis versus controls and four mRNAs associated with disease progression. Our integrated model emphasized the prominence of the PI3K/AKT1 pathway, which is important in T cell and mTOR function. Novel immune related genes and miRNAs including *LYST*, *RGS14*, *SLFN12L*, and hsa-miR-199b-5p, distinguished sarcoidosis from controls. Our integrated model also demonstrated differential expression/methylation of *IL20RB*,* ABCC11*,* SFSWAP*, *AGBL4*, miR-146a-3p, and miR-378b between non-progressive and progressive sarcoidosis.

**Conclusions:**

Leveraging the DNA methylome, transcriptome, and miRNA-sequencing in sarcoidosis BAL cells, we detected widespread molecular changes associated with disease, many which are involved in immune response. These molecules may serve as diagnostic/prognostic biomarkers and/or drug targets, although future testing is required for confirmation.

**Supplementary Information:**

The online version contains supplementary material available at 10.1186/s12931-024-02919-7.

## Introduction

Sarcoidosis is a heterogeneous disease characterized by non-caseating granulomatous inflammation that differentially impacts Black individuals and women [1]. The lungs are involved in over 90% of individuals [2]. Those with pulmonary sarcoidosis can be asymptomatic or demonstrate remission / resolution; however, progression can result in impairment and/or pulmonary fibrosis, the main cause of mortality [3]. The course of pulmonary sarcoidosis is unpredictable, with at least 25% of patients developing chronic or progressive disease requiring treatment [4]. Driven by a combination of genetic, environmental, and host immunologic factors, the underlying cause(s) of sarcoidosis are currently unknown. Multiple lines of evidence point towards an antigenic stimulus including HLA allele associations, environmental, seasonal, and regional patterns, and a Th1 predominate immune response in which CD4 + T cells secrete IFN-γ and TNF-α [5]. In addition, aberrant and dysfunctional immune responses are associated with sarcoidosis and supported by genome wide transcriptome studies [6]. While previous studies have elucidated many contributors to disease development and progression, many knowledge gaps remain.

Epigenetic mechanisms such as DNA methylation (DNAm) and microRNAs (miRNAs) mediate gene expression, are modified by exposures, and are dynamic and reversible, making them candidates for gene regulation in sarcoidosis as well as promising biomarkers and therapeutic targets. Epigenetic dysregulation has been identified in many lung diseases and likely drives sarcoidosis progression and manifestations. We have previously demonstrated changes in DNAm and mRNA gene expression in bronchoalveolar lavage (BAL) cells from chronic beryllium disease (a granulomatous lung disease caused by beryllium exposure) patients and a small sample of sarcoidosis patients [7, 8]. No other studies have evaluated epigenome-wide DNAm in sarcoidosis although others have identified miRNAs associated with disease [9, 10]. Additionally, BALF miRNAs such as miR-27b, miR-192, and miR-221 have been associated with pulmonary sarcoidosis progression [11]. These studies were limited by small sample sizes, targeted as opposed to genome-wide approaches, and a lack of integration of different omics modalities.

With a larger integrated approach utilizing several genomic data types, we hypothesize that epigenomic studies could link risk factors to disease pathobiology to better understand disease course, and subclassify patients based on molecular profiling; ultimately this would direct focused research on disease manifestations and treatment. In a first step, we conducted this study to profile genome-wide DNAm, mRNA and miRNA expression in sarcoidosis BAL cells, stratified by disease progression. Analyzing each dataset separately, we identified many molecular features associated with disease overall. By next constructing a sparse multi-omic model incorporating DNAm, mRNAs, and miRNAs, we identified features associated with sarcoidosis and pulmonary progression.

## Methods

### Study population

Sarcoidosis BAL cell samples were obtained from the Genomic Research in Alpha-1 Antitrypsin Deficiency and Sarcoidosis (GRADS) consortium [12] including National Jewish Health (NJH, *n* = 17) and non-NJH GRADS cases (*n* = 39) and cases from the Granuloma Biorepository at NJH (*n* = eight). Controls with no history of lung disease were obtained from the NJH Donor Lung Core (*n* = 16). All sarcoidosis subjects met the ATS/ERS criteria [13] for tissue biopsy confirmation of diagnosis of sarcoidosis. For all NJH cases, medical records were reviewed for clinical features including acuity of presentation (acute/non-acute), organ involvement, pulmonary function tests (PFT), chest imaging and immunosuppressive treatment at time of BAL and up to two years after BAL. For the non-NJH GRADS cases, acuity of presentation, PFTs, and immunosuppressive treatment were available at and up to six months after BAL. Cases with fibrotic/Scadding stage 4 chest radiographic disease and/or on immunosuppressive treatment at time of BAL were excluded.

Sarcoidosis cases were categorized as non-progressive or progressive pulmonary phenotypes. The non-progressive phenotype was defined as having either acute (i.e. consistent with Lofgren’s syndrome) or non-acute disease presentation, no new organ involvement, lung function testing with < 10% decline in FVC or FEV_1_, < 15% decline in DLCO, and stable chest imaging within two years after BAL. The progressive phenotype had a non-acute disease presentation; lung function testing with ≥ 10% decline in FVC or FEV_1_; or ≥ 15% decline in DLCO; worsening chest imaging; and/or required initiation of systemic immunosuppressive treatment any time up to two years after BAL. Non-NJH GRADS cases were phenotyped based on disease acuity, PFT, and treatment status only.

### Bronchoscopy and nucleic acid processing

Bronchoscopy with BAL was performed as previously described [12, 14]. Cells were isolated and frozen at -80 C in RLT buffer. DNA and RNA were extracted using the Qiagen AllPrep DNA/RNA extraction mini kit. Purified genomic DNA was bisulfite-converted with the Zymo EZ-96 DNA Methylation bisulfite conversion kit, followed by whole-genome amplification and enzymatic fragmentation. DNA was denatured and hybridized to Illumina Infinium HumanMethylationEPIC BeadChips, followed by single base extension. Hybridized BeadChips were stained, washed, and scanned using Illumina’s iScan System. mRNA libraries were prepared from 500 ng total RNA with TruSeq stranded mRNA library preparation kits (Illumina) and miRNA libraries were constructed using Lexogen Small RNA-Seq library preparation kits. RNA libraries were sequenced at an average depth of 80M 150 bp paired-end reads on the Illumina NovaSeq 6000.

### Data analysis

RNA-Sequencing data was previously produced on 28 samples by the GRADS consortium [6]. RNA paired-end reads from both GRADS and NJH samples were aligned at the gene level to Ensembl GrCh38 using STAR [15]. The forward read of miRNA FASTQ files were aligned to sequences from miRBase v22.1 [16–21] using miR-MaGiC [22]. miRNAs present in at least 50% of samples were retained (*n* = 818). Illumina idat signal intensity files were processed using SeSAMe; within-sample normalization with out-of-band probes and dye bias correction were performed [23]. Probes with non-unique mapping and off-target hybridization were removed. Additionally, probes with an average detection *p-*value ≥ 0.05 within samples (indicating an inability to statistically discern signal from background noise) and sex chromosome probes were removed prior to analysis. Methylation levels were analyzed as M values, and are presented in results as β values [24].

For each dataset, linear models were fit to each feature testing for an effect of sarcoidosis diagnosis or progressive vs. non-progressive disease while adjusting for age, sex, and smoking status (using the R package limma [25] for methylation data and DESeq2 [26] for RNA datasets). We further adjusted for three principal components derived from the data in methylation and mRNA case-control comparisons. Shrunken effect sizes from RNA comparisons (expressed as log_2_(fold change)) were calculated using Approximate Posterior Estimation for the GLM (apeglm) [27]. Null test statistic distributions were derived for each analysis using bacon to reduce bias and inflation [28]. *P*-values were adjusted to a 5% false discovery rate (FDR) to account for multiple testing using the Benjamini-Hochberg procedure [29]. Enrichment of significant results in the Gene Ontology (GO) resource [30] and the Kyoto Encyclopedia of Genes and Genomes (KEGG) [31] was performed using GOmeth [32] for DNAm data and clusterProfiler [33] for mRNA data. CpGs were annotated to CpG islands, shelves, and shores, and gene elements using the annotatr R package [34]. Experimentally confirmed miRNA target genes were obtained from MirTarBase [35]. For each miRNA, we retained target genes that had at least 2 independent sources of experimental evidence of association with the relevant miRNA, including at least one source using assays considered strong evidence by MirTarBase (reporter assay, Western blot, or qPCR).

### Data integration analysis for biomarker discovery using latent cOmponents (DIABLO)

Methylation M values and normalized mRNA and miRNA gene expression values were used as input for a DIABLO [36] model implemented in the mixOmics R package [37]. Gene expression values were normalized to library size using DESeq2 [26] and transformed to account for heteroscedasticity with a variance stabilizing transformation (VST) [38]. A subset of features from each dataset for two model components were selected for model input using LASSO regression and five-fold cross-validation was repeated ten times.

## Results

### Demographics of study population

We recruited 64 sarcoidosis cases, including 26 progressive and 38 non-progressive, and 16 healthy controls. Demographic information at time of BAL is displayed in Table 1. No significant differences were observed in race, ethnicity, or age. Non-progressive cases were more likely female than progressive cases. Progressive cases presented with significantly reduced FEV_1_ and FVC and more Stage 2 disease.

### Immune gene expression differs by disease status and to a lesser degree by disease progression

**Fig. 1:**
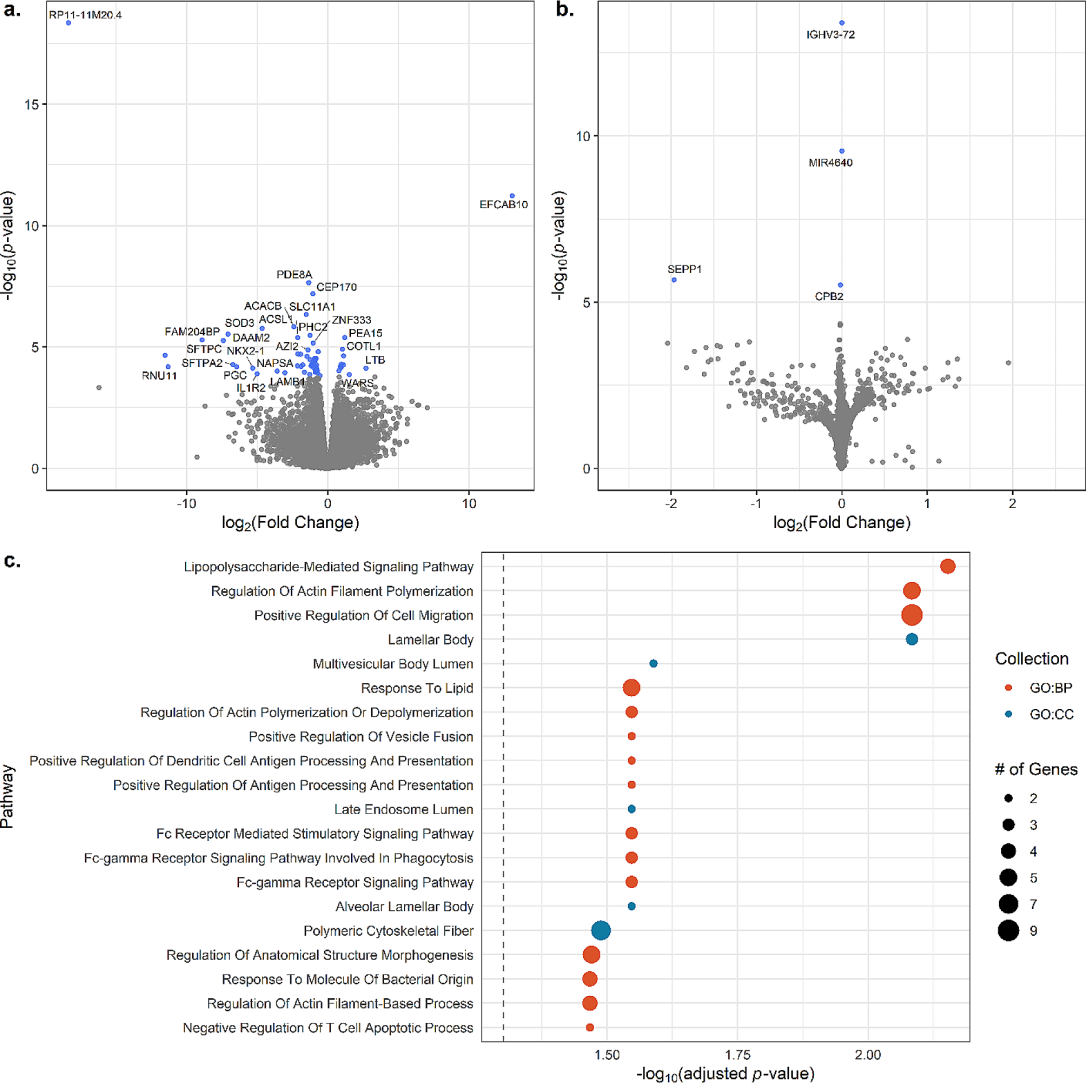
Differentially expressed genes in sarcoidosis. **A**) Differentially expressed genes in sarcoidosis vs. controls. Significant genes at an FDR-adjusted *p*-value threshold of 5% are colored in blue. Unadjusted *p*-values are plotted on the y-axis. **B**) Differentially expressed genes in progressive vs. non-progressive sarcoidosis. Significant genes at an FDR-adjusted *p*-value threshold of 5% are colored in blue. Unadjusted *p*-values are plotted on the y-axis. **C**) Pathway enrichment of mRNAs. The gray dashed line indicates an FDR-adjusted *p*-value threshold of 5%. GO: BP: Gene Ontology Biological Process, GO: CC: Gene Ontology Cellular Component

We analyzed gene expression data to assess differences between both cases and controls and progressive and non-progressive disease. In the sarcoidosis-control comparison, we detected 1,842 differentially expressed genes (DEGs), of which 379 (20.6%) were significantly increased in sarcoidosis (Supplementary Figure S1; Supplementary Table S1A). Due to *p-value* inflation (λ = 1.558) and a skewed test-statistic distribution (Supplementary Figure S1), we ran an additional model adjusting for three principal components to improve the model fit; this resulted in 64 DEGs that meet the FDR-adjusted *p* < 0.05 and an additional 61 at FDR-adjusted *p* < 0.1 (Fig. 1A; Supplementary Table S1B). Many of the significant genes from this model are also included in the 1,842 DEGs (20/64; 31.3%). Top significant upregulated genes involved many genes relating to immunity, including cytokine and chemokine signaling (chemokine *CCL5/RANTES*, chemokine receptor *CXCR3*, *IL16*), T lymphocytes (*CD6*, *IL16*, *LTB*), and HLA class II. Most significant downregulated DEGs include surfactant genes *SFTPA2* and *SFTPC*, and transcription factors important in lung cell specification such as *CEBPD* (CCAAT Enhancer Binding Protein Delta) and *NKX2-1* (NK2 homeobox 1). Four DEGs (*IGHV3-72*,* MIR4640*,* SEPP1*, & *CPB2*) were found in progressive vs. non-progressive cases, although all except *SEPP1* displayed small effect sizes (|log_2_(fold change)| < 0.01) driven by low expression (Fig. 1B).

We next tested whether 125 sarcoidosis DEGs (FDR-adjusted *p-*value < = 0.1) were overrepresented in GO [30] and KEGG pathways using clusterProfiler [33] (Fig. 1C; Supplementary Table S2). Significant genes were enriched for 29 GO and one KEGG pathways. The most significantly enriched GO terms include lipopolysaccharide-mediated signaling pathway, regulation of actin filament polymerization, and positive regulation of cell migration. The significant KEGG pathway was epithelial cell signaling in Helicobacter pylori infection.

### DNA methylation differs between cases and controls and overlaps with gene expression

**Fig. 2:**
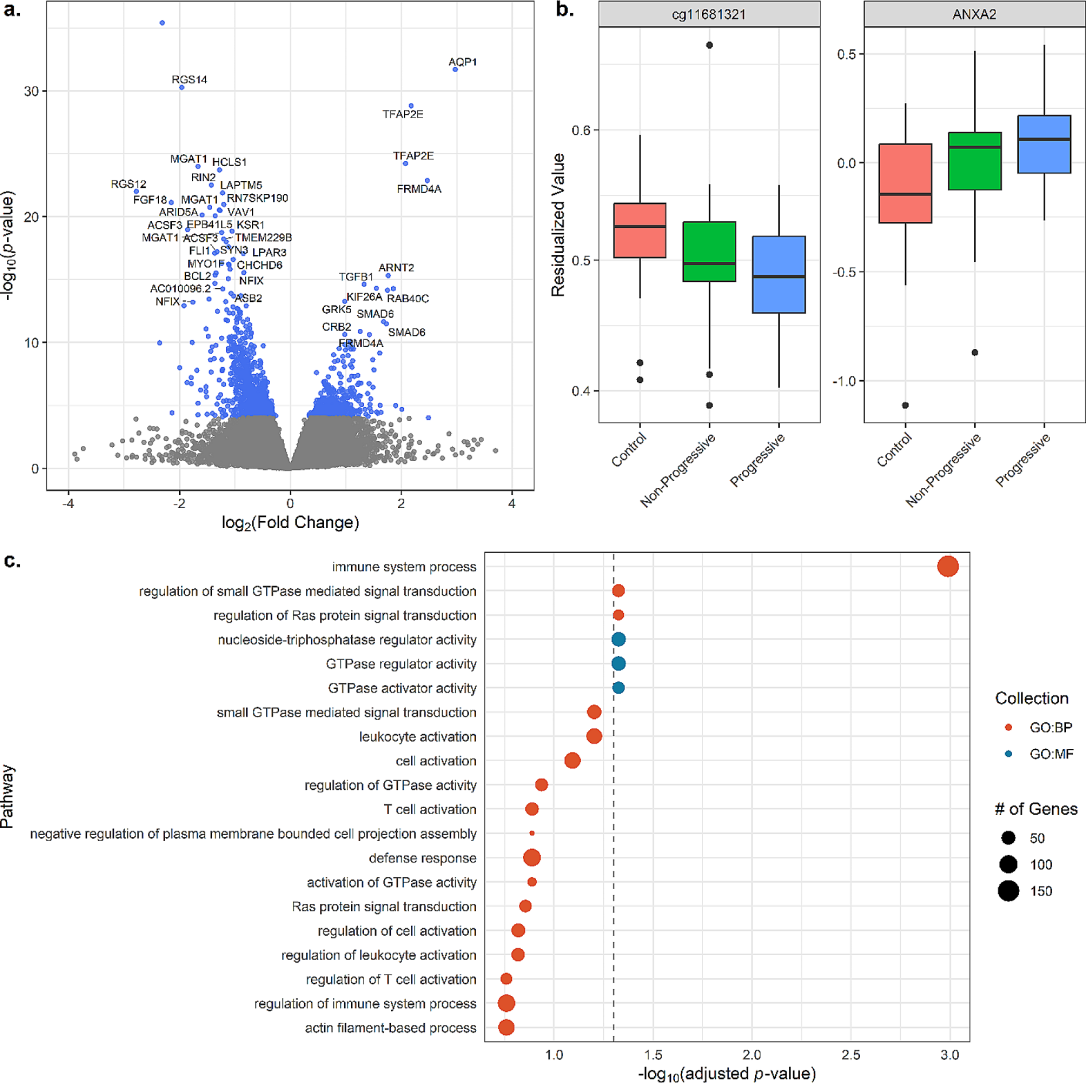
Differentially methylated sites in sarcoidosis. **A**) Differentially methylated sites in sarcoidosis vs. controls. Significant sites at an FDR-adjusted *p*-value threshold of 5% are colored in blue. Unadjusted *p*-values are plotted on the y-axis. Sites are labeled by associated gene. **B**) Boxplots of *ANXA2* cg11681321 methylation and expression by sample group. Omics values were residualized by age, sex, and three principal components from the relevant data type. **C**) Pathway enrichment of hypomethylated DNAm sites. The gray dashed line indicates an FDR-adjusted *p*-value threshold of 5%. GO: BP: Gene Ontology Biological Process, GO: MF: Gene Ontology Molecular Function

We next tested for differential methylation (DM) and identified 46,812 CpG sites associated with sarcoidosis, of which 38,504 map to 15,208 unique genes (Supplementary Table S3A). As with mRNA, due to poor model fit (Supplementary Figure S2), we ran an additional model adjusting for three principal components to improve the model fit; this resulted in 1,459 CpGs that meet the FDR-adjusted *p* < 0.05 (Fig. 2A; Supplementary Table 3B). 897 (61.4%) of CpGs are shared with the model without PCs. We did not detect any DNAm sites significantly associated with disease progression. The majority of significant CpGs were hypomethylated in sarcoidosis (948; 64.9%). Significant probes were enriched for intronic regions (66.7% vs. 60.6%; Fisher’s exact test *p*-value = 2.0 × 10^− 6^) and FANTOM5 enhancers (15.7% vs. 4.10%; Fisher’s exact test *p*-value = 1.5 × 10^− 66^) relative to all tested DNAm sites. Hypomethylated DM sites were significantly enriched for six GO terms, with top hits including immune system process, GTPase activator activity, and GTPase regulator activity (Fig. 2C; Supplementary Table S4). The most significant hypomethylated DM sites include CpGs that map to an intergenic region 17 kb upstream of CCR7 and a promoter of RGS14 as well as an alternative promoter of *MGAT1*. Top hypermethylated DM sites mapped to the first exon of *AQP1* and an exon of *TFAP2E*.

We next overlapped DEGs and differentially methylated probes (DMPs) in sarcoidosis vs. controls based on gene ID. A single gene, *ANXA2*, showed evidence of both differential methylation and expression (Fig. 2B). After regressing out covariates, *ANXA2* displays increased expression in cases as well as hypomethylation of cg11681321, a CpG within an intron of *ANXA2*.

### miRNA expression differs between cases and controls and targets DEGs

**Fig. 3:**
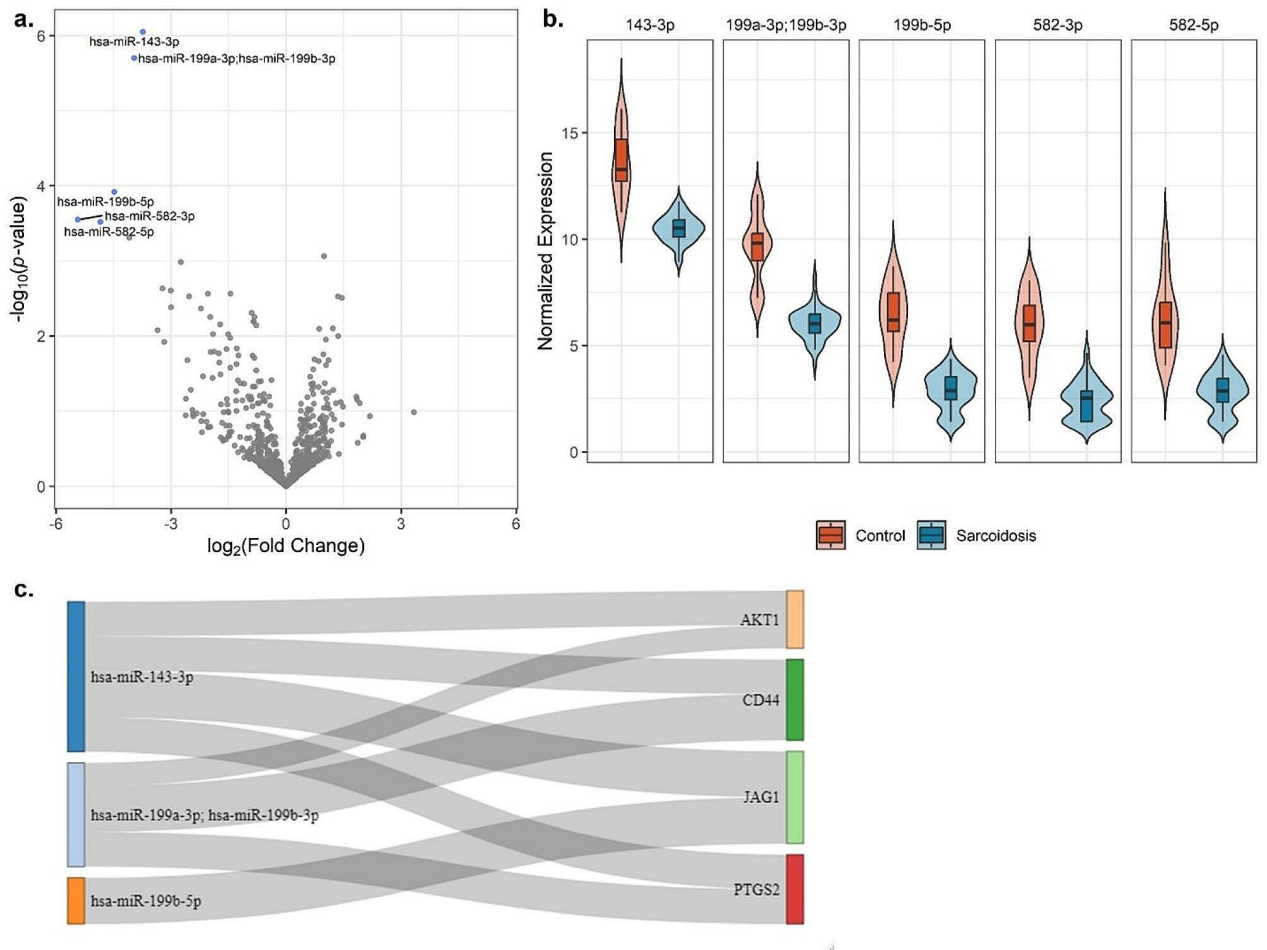
Differentially expressed microRNAs in sarcoidosis. **A**) Differentially expressed miRNAs in sarcoidosis vs. controls. Significant genes at an FDR-adjusted *p*-value threshold of 5% are colored in blue. Unadjusted *p*-values are plotted on the y-axis. **B**) Distribution of significant miRNAs’ expression in cases and controls. **C**) Sankey plot connecting DE miRNAs to target genes targeted by > 1 DE miRNA. Connection width represents number of sources confirming relationship

We next compared miRNA expression in sarcoidosis vs. controls and identified five miRNAs (hsa-miR-143-3p, hsa-miR-199a-3p/hsa-miR-199b-3p, hsa-miR-199b-5p, hsa-miR-582-3p & hsa-miR-582-5p) downregulated in sarcoidosis (Fig. 3A-B; Supplementary Table S5). No miRNAs were associated with progression. We derived experimentally validated target genes for each significant miRNA using MirTarBase [35]. We identified 67 target genes, of which four (*AKT1*,* CD44*,* JAG1*,* PTGS2*) are targeted by two DE miRNAs (Fig. 3C).

### Integrated model reveals disease and progression associations not found in individual analyses

**Fig. 4:**
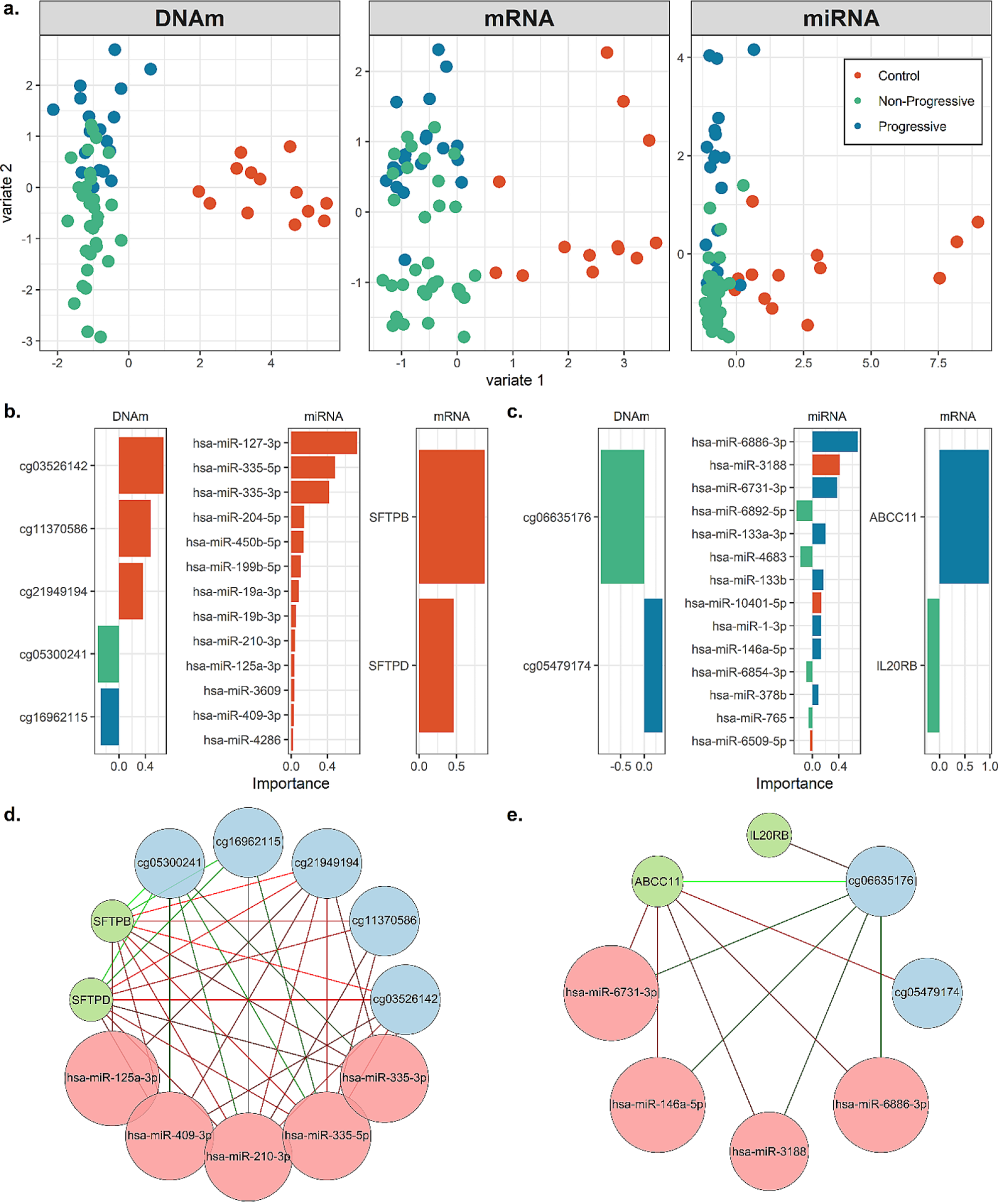
A sparse multi-omic model of sarcoidosis and progression. **A**) Projection of samples based on selected feature weights. Points represent samples, colored by disease group, and are plotted for each data type on the top latent variables from the DIABLO multi-omic model. **B**) Feature importance of selected features for latent variable (1) Bars are colored by strongest sample group loading. **C**) Feature importance of selected features for latent variable (2) Bars are colored by strongest sample group loading. **D**) Network constructed from feature correlations on latent variable (1) Features are colored by dataset. Edges are colored by feature correlation, with green representing positive correlation. Features in the network share at least 90% correlation. **E**) Network constructed from feature correlations on latent variable (2) Features are colored by dataset. Edges are colored by feature correlation, with green representing positive correlation. Features in the network share at least 50% correlation

We used DIABLO to integrate the three datasets, including individuals present in all datasets after QC (*n* = 65; 13 controls, 19 progressive, 33 non-progressive sarcoidosis). With a goal to determine molecular features separating three groups (controls, progressive, and non-progressive sarcoidosis), we constructed a multi-omic model with two latent variables, which are linear combinations of input features. Using 5-fold cross validation repeated ten times, we determined that selecting five DNAm sites, two mRNAs, and 13 miRNAs for latent variable 1 and two DNAm sites, two mRNAs, and 14 miRNAs for latent variable 2 maximized the prediction accuracy of the model and resulted in clustering based on diagnosis (Fig. 4A; Supplementary Table S6). Latent variable 1 separates controls from sarcoidosis samples and latent variable 2 separates progressive from non-progressive sarcoidosis. Features included in each latent variable are shown in Fig. 4B; Supplementary Table S7. We further constructed networks based on correlations of feature weights for each latent variable (Fig. 4C). The two mRNAs contributing to latent variable 1 were *SFTPB* and *SFTPD*. DNAm sites contributing to latent variable 1 include DNAm sites hypermethylated in cases cg16962115, within an intron of *LYST*, and cg05300241 within an intron of *PIK3CD*. The remaining three DNAm sites were hypomethylated and included cg21949194 within an enhancer of *SOS1* (SOS Ras/Rac guanine nucleotide exchange factor 1), cg11370586 within the predicted promoter of *RGS14*, and cg03526142 within an exon of *SLFN12L*. All DNAm sites contributing to latent variable 1 were DMPs in our previous methylation analysis. In addition to the DE miR hsa-miR-199b-5p, miRNA features on latent variable 1 include hsa-miR-204-5p. Features contributing to latent variable 2 include the DNAm sites cg05479174, within an exon of *SFSWAP*, and cg06635176 within an intron of *AGBL4*. *IL20RB* (interleukin 20 receptor subunit beta) mRNA was upregulated in non-progressive sarcoidosis, while *ABCC11* (ATP binding cassette subfamily C member 11) expression was higher in progressive sarcoidosis. miRNAs contributing to latent variable 1 include hsa-miR-146a-3p and hsa-miR-378b.

## Discussion

In this study, we present the first application of multi-omic integration in sarcoidosis, leveraging coding and miRNA expression with DNA methylation data to construct a multiomic network. Through initial genome-wide profiling of DNA methylation, mRNA expression, and miRNA expression in sarcoidosis BAL, we identified 64 DEGs, 1,459 DMPs, and five DE miRNAs in sarcoidosis relative to healthy controls, as well as four DEGs associated with sarcoidosis progression. By integrating omic datasets, we define pathogenic molecules/genes in sarcoidosis not identified using conventional modeling methods in single-omics datasets. While our single-omics approach only demonstrated four DEGs for progression, the multiomic model identified several progression-associated methylation, mRNA, and miRNA features. We identify previously reported molecules and pathways associated with disease as well as implicate novel molecular features as potential drivers and modifiers of sarcoidosis, thus demonstrating the potential of integrative approaches.

Our single-omic analyses implicate multiple genes in the pathogenesis of sarcoidosis, with many of these genes involved in processes relating to inflammation and immunity (potentially associated with those involved in viral/atypical bacterial infections and autoimmunity) and extracellular matrix signaling. While we detected many associations with case status, we detected few omic associations with progression. We identified four mRNAs associated with progression, including *SEPP1.* Results from the GRADS study demonstrated that *SEPP1* levels were inversely correlated with DLCO (%) and FVC (% pred) in sarcoidosis individuals [6]. These finding support a progressive phenotype, including lung function changes that we and others have used in the definition of progressive pulmonary disease.

DNAm and mRNA association analyses both identified differential regulation of the gene *ANXA2*, which displays hypomethylation and increased gene expression in sarcoidosis samples. ANXA2 protein levels have previously been reported to be increased in BALF from sarcoidosis cases [39]. In addition, ANXA2 is found on macrophages, as a cytoplasmic and cell surface molecule that is involved in endocytosis, a pathway frequently found associated with sarcoidosis; however the exact role of this gene in sarcoidosis and/or progression is unclear at this time and will require future study.

Our study also demonstrated five DE miRNAs downregulated in sarcoidosis cases in the first study of genome-wide miRNAs in sarcoidosis. While these associations are novel, multiple target genes of these miRNAs have been implicated in sarcoidosis previously, including the genes *AKT1*, *CD44*,* JAG1*, and *PTGS2*. For example, CD44 has been found in areas of granuloma formation and fibrosis [40] and is differentially expressed between Lofgren syndrome versus non-Lofgren syndrome subjects [41].

Our integrated model revealed several novel genes and miRNAs between sarcoidosis and controls, including *LYST*, *RGS14*, *SLFN12L*, and hsa-miR-199b-5p. The first three genes appear to have important roles in immune function. Specifically, *LYST*, a regulator of endosome/lysosome trafficking, can regulate TLR3 and TLR4 mediated pathways [42], genes involved in the innate immune system which have been implicated in sarcoidosis [6, 43, 44]. *RGS14* is expressed in lymphocytes and regulates chemokine receptors to control immune responses to exogenous agents [45]. Finally, *SLFN21L* regulates thymocyte development and is downregulated in T-cell activation, suggesting a role as an immune response regulator [46].

Our integrated analyses demonstrated miRNAs DE between progressive/non-progressive sarcoidosis including those previously described. Specifically, miR-146a-3p upregulated in progressive sarcoidosis is an indicator of inflammation and oxidative stress that may target *TLR4* and was previously found elevated in sarcoidosis BALF, as well as serum [47, 48]. miR-378b, which was upregulated in progressive cases, was previously found associated with sarcoidosis [49]. We also identified novel genes including *IL20RB*,* ABCC11*,* SFSWAP* and *AGBL4.* Interestingly, *IL20RB* expression was increased in non-progressive versus progressive sarcoidosis in our study, suggesting that the activation of this inflammatory pathway is increased in the non-progressive phenotype. *ABCC11* is a gene that influences macrophage differentiation and induces TNF-α and IL17 through TLR4 signaling [50]; these results as well as the novel findings above support the importance of innate immune response genes in sarcoidosis.

Both our individual and integrative analyses identify molecules involved in PI3K/AK1 signaling, a pathway already recognized in sarcoidosis pathogenesis. For example, we observe hypermethylation of *PI3KCD* (phosphatidylinositol-4,5-bisphosphate 3-kinase catalytic subunit delta), which encodes a component of PI3K, and complexes with AKT1 to impact T cell differentiation and function [51]. Our miRNA results demonstrate *AKT1* as a target of downregulated miRNAs (miR-143-3p and miR-199a/miR-199b) in sarcoidosis. Both downregulation and upregulation of the PI3K/AKT signaling pathways have been associated with sarcoidosis [39, 51]. Previous studies demonstrated both reduced proliferative response and exhaustion of T cells in progressive sarcoidosis is thought to be driven in part by inhibition of PI3K/AKT1 signaling [51]. The PI3K/AKT1 pathway has also been implicated in activation of mTOR [39], which has been associated with granuloma formation [52]. Interestingly, in the recent GRADS study, PI3K activation was associated with an endotype of sarcoidosis characterized by hilar lymphadenopathy, pulmonary reticulation, less multiorgan involvement, and more environmental associations [6].

While our sample size is larger than most previous sarcoidosis omic studies, power in our analyses was limited, especially in our phenotype analyses. In future studies, it will be important to both increase sample sizes and investigate associations with better-powered quantitative measures related to disease such as pulmonary function testing variables. We were unable to correct for cell proportions in sarcoidosis subjects relative to controls as controls lacked cell differentials. Despite this, final case-control models (adjusting for principal components in the mRNA and DNAm comparisons) display minimal test-statistic inflation (λ = 1.09–1.31). Altered cell proportions may explain the downregulated epithelial genes in sarcoidosis BAL (e.g. decreased surfactant proteins in sarcoidosis) and some of the DNAm sites and DEGs detected in sarcoidosis versus control comparisons, although in general epithelial cells are not a large proportion of BAL. Finally, our multi-omic models did not take into account demographic information. Regressing out demographic information such as age and sex may lead to more predictive networks. Despite these shortcomings, we detected widespread molecular changes and constructed multi-omic networks associated with sarcoidosis as well as progression.

## Conclusions

Using integrative methods, we identified DNA methylation, mRNA, and microRNA associations with sarcoidosis and disease progression in bronchoalveolar lavage cells. Some of these molecular markers have been identified previously in the literature, while others are novel. Molecules discovered in these analyses shed light on disease pathogenesis and may also be leveraged therapeutically or as biomarkers after replication/validation in additional populations.

**Table 1:** Demographic information of control, progressive sarcoidosis, and non-progressive sarcoidosis cases at time of bronchoscopy with lavage

	Control	Non-Progressive	Progressive	*p*
n	16	38	26	
Age (mean (SD))	55.62 (9.74)	51.68 (9.88)	51.19 (9.61)	0.316*
Sex = Male (%)	11 (68.8)	11 (28.9)	16 (61.5)	0.006**
Race (%)				0.242**
Asian	1 (6.2)	1 (2.6)	0 (0.0)	
Black	0 (0.0)	6 (15.8)	5 (19.2)	
White	15 (93.8)	31 (81.6)	21 (80.8)	
Ethnicity = Non-Hispanic (%)	15 (93.8)	36 (94.7)	25 (100.0)	0.441**
Smoking Status = Former (%)	0 (0.0)	12 (31.6)	6 (23.1)	0.0275**
FVC (mean (SD))		97.42 (11.25)	85.92 (14.21)	0.001^#^
FEV_1_ (mean (SD))		101.03 (12.19)	86.40 (15.04)	< 0.001*
DLCO (mean (SD))		87.71 (21.81)	84.42 (12.64)	0.491*
Scadding Stage (%)				< 0.001**
0		13 (34.2)	1 (3.8)	
1		20 (52.6)	1 (3.8)	
2		4 (10.5)	22 (84.6)	
3		1 (2.6)	2 (7.7)	

*One-way ANOVA, **Fisher’s exact test, ^#^Mann-Whitney U test. FVC: forced vital capacity. FEV_1_: forced expiratory volume over 1 s. DLCO: diffusing capacity of the lung for carbon monoxide

### Electronic supplementary material

Below is the link to the electronic supplementary material.

Supplementary Material 1

Supplementary Material 2

Supplementary Material 3

Supplementary Material 4

Supplementary Material 5

Supplementary Material 6

Supplementary Material 7

Supplementary Material 8

Supplementary Material 9

## Data Availability

Data generated as a part of this study (DNAm, miRNA, and mRNA datasets, as well as relevant deidentified phenotypic data) have been deposited in the National Center for Biotechnology Information Gene Expression Omnibus. IDAT files and a processed matrix of DNAm data are available through accession GSE263432, a counts table of mRNA data is available through GSE263448, and a counts table of miRNA data is available through GSE263451.
