# Supplementary figures and images for: Multi-omic signatures of sarcoidosis and progression in bronchoalveolar lavage cells
Source: Respir Res. 2024 Jul 30;25:289. doi: 10.1186/s12931-024-02919-7 (PMC11290275; doi:10.1186/s12931-024-02919-7)

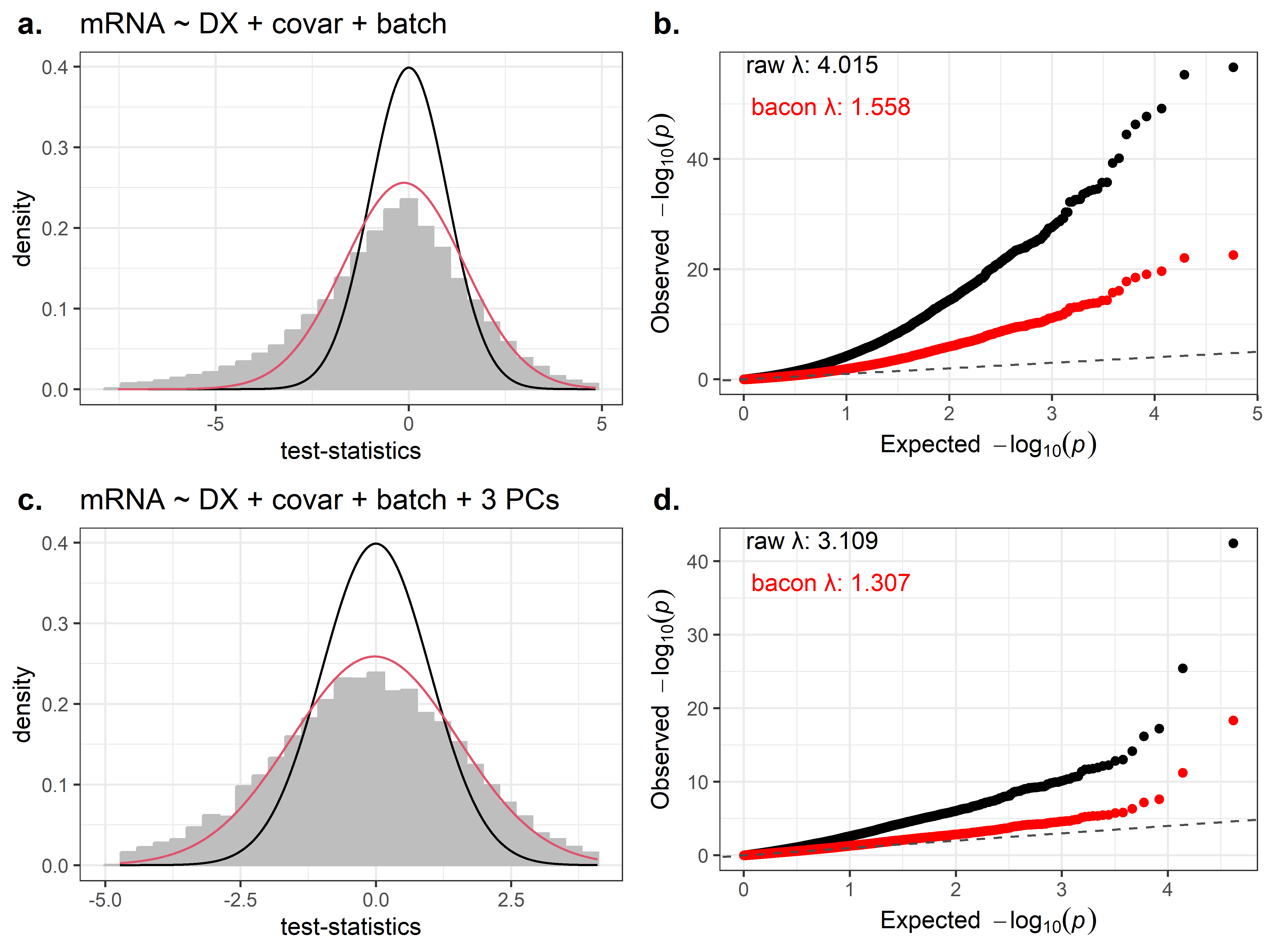

Supplement: Supplementary file 1 — Supplementary Material 1 [file 12931_2024_2919_MOESM1_ESM.png]

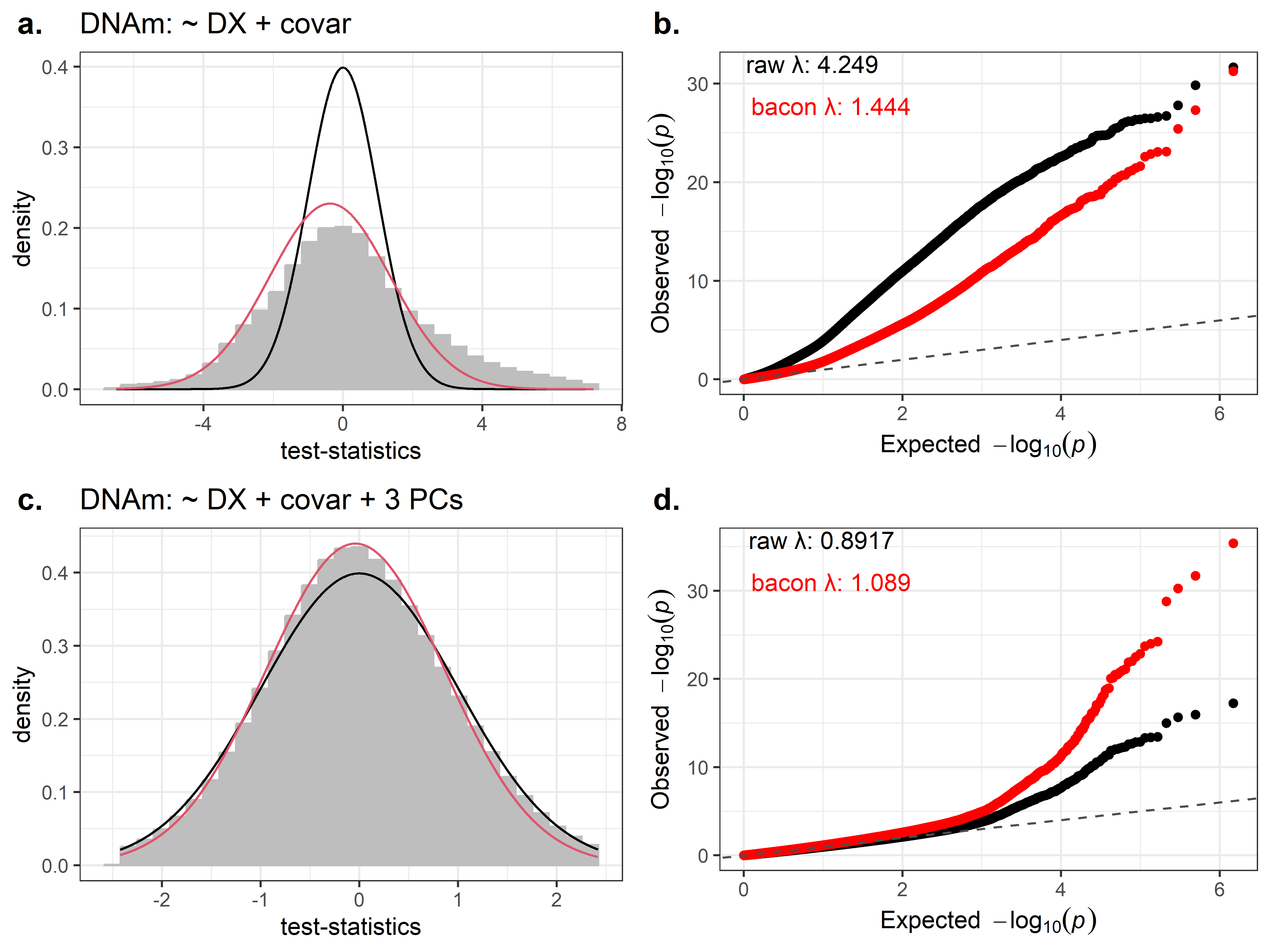

Supplement: Supplementary file 2 — Supplementary Material 2 [file 12931_2024_2919_MOESM2_ESM.png]
